# Y-Box Binding Protein 1 Expression in Trophoblast Cells Promotes Fetal and Placental Development

**DOI:** 10.3390/cells9091942

**Published:** 2020-08-22

**Authors:** Nicole Meyer, Anne Schumacher, Urs Coenen, Katja Woidacki, Hannah Schmidt, Jonathan A. Lindquist, Peter R. Mertens, Ana C. Zenclussen

**Affiliations:** 1 Experimental Obstetrics and Gynecology, Medical Faculty, Otto-von-Guericke University, 39108 Magdeburg, Germany; nicole.meyer@med.ovgu.de (N.M.); anne.schumacher@med.ovgu.de (A.S.); urs.coenen@outlook.com (U.C.); katja.woidacki@med.ovgu.de (K.W.); Hannah.Schmidt.HS@posteo.de (H.S.); 2 Department of Environmental Immunology, Helmholtz Centre for Environmental Research, 04318 Leipzig, Germany; 3 Department of Nephrology and Hypertension, Diabetes and Endocrinology, Otto-von-Guericke University, 39120 Magdeburg, Germany; jon.lindquist@med.ovgu.de (J.A.L.); peter.mertens@med.ovgu.de (P.R.M.)

**Keywords:** Y-box binding protein 1, trophoblast, fetal and placental development, pregnancy, serial high-frequency ultrasonography

## Abstract

Y-box binding protein 1 (YB-1) is pivotal for the regulation of cancerogenesis and inflammation. However, its involvement in pregnancy processes such as fetal and placental development remains to be elucidated. We studied *Ybx1* (YB-1)^+/−^ heterozygous intercrossings and compared them to YB-1^+/+^ wild-type (WT) combinations. Additionally, we generated trophoblast-specific YB-1-deficient mice by pairing FVB Cyp19-Cre females to YB-1^fl/fl^ males. YB-1^fl/fl^-paired FVB WT females served as controls. Serial in vivo ultrasound measurements were performed to assess fetal and placental parameters. After sacrificing the females, implantation and abortion rates were recorded, spiral artery (SA) remodeling was analyzed and fetal and placental weights were determined. Compared to YB-1^+/+^ counterparts, YB-1^+/−^ females showed reduced implantation areas at gestation day (GD)10, insufficiently remodeled SAs at GD12, increased placental diameter/thickness ratios at GD14 and reduced placental and fetal weights at GD14. Compared to WT, Cyp19-Cre females with YB-1-deficient placentas showed reduced implantation areas at GD8, 10 and 12; decreased placental areas and diameters at GD10 and 12; diminished placental thicknesses at GD12; as well as reduced placental weights at GD12 and 14. In conclusion, our data suggest haploinsufficiency of YB-1 resulting in disturbed fetal and placental development. Moreover, we provide the first evidence for the relevance of trophoblast-specific YB-1 for placentation.

## 1. Introduction

Y-box binding protein 1 (YB-1) is a multifunctional cold shock protein that regulates a variety of biological processes including transcriptional and translational control at cellular level. In the nucleus, YB-1 participates in DNA repair and replication mechanisms as well as in gene transcription. In the cytoplasm, YB-1 regulates mRNA translation and protects the mRNA from degradation. A detailed overview of YB-1 functions is provided in Reference [1]. Moreover, in response to inflammatory stimuli and oxidative insults, YB-1 is actively secreted by different cell types and exerts its autocrine and paracrine effects on various target cells [2,3]. 

Pregnancy is arguably the most critical period in every individual’s life, as the events occurring in the maternal womb shape adult health [4,5]. During this period, timely and finely regulated interactions between the maternal and fetal tissues not only guarantee the survival of the genetically foreign fetus, but also actively support its adequate development. The complex network between maternal and fetal structures is built up by a plethora of molecules whose expression is strictly controlled at distinct gestational ages. Thus, knowing the pivotal functions of YB-1 in regulating gene expression, it is conceivable that YB-1 plays a major role in fundamental pregnancy processes, such as fetal and placental development. Notably, YB-1 is expressed during almost the entire ontogenesis, particularly in its early stages [6]. Its presence has been proven in mouse embryonic stem cells [7], and embryonic day (E)13.5 embryos show an almost ubiquitous expression of YB-1 with the highest expression in the central nervous system, lung, kidney and heart [8]. In 2005, Lu and colleagues were the first to develop an YB-1 loss-of-function mouse model and provide evidence for a nonredundant role of YB-1 in late embryonic development and perinatal survival of the pups. These authors showed that most *Ybx1* (from now on, YB-1) -knockout (KO) embryos developed normally until E13.5, but exhibited severe growth retardation and mortality thereafter. Only a few YB-1 KO fetuses were born, albeit they died shortly after birth. Detailed analyses of the KO embryos revealed neurological lesions, severe hemorrhages and respiratory failures, and it was suggested that these malformations at least partially accounted for the perinatal lethality [9]. One year later, Uchiumi and colleagues also performed YB-1 heterozygous intercrossings and found YB-1 KO embryos to be growth-retarded as early as E10.5. Furthermore, the authors showed that YB-1-deficient embryos die between E14.5 and E18.5. In their hands, YB-1 KO embryos showed hemorrhages, severe anemias and severe brain malformations, but were otherwise normal in appearance [8]. As YB-1 is a key molecule in transcriptional and translational regulation, both research groups assumed changes in the transcriptome and proteome of embryonic fibroblasts from YB-1-deficient embryos. Remarkably, YB-1-deficient embryonic fibroblasts did not exhibit major changes in either cellular process, but showed an enhanced sensitivity to different stressors as well as a reduced ability to grow and divide [8,9]. 

It has been speculated that late embryonic hypoplasia as consequence of an YB-1 deficiency might derive from a placental defect [10]. Although no detailed analysis regarding placental structure and function was performed, it was obvious that YB-1 KO placentas were smaller than YB-1-sufficient placentas [9]. A smaller placental volume often goes hand in hand with suboptimal function; this can in turn impair fetal growth, wellbeing and survival. Therefore, it is imperative to address this issue in more detail to understand the possible implications in pregnancy complications and suboptimal fetal development. 

For certain cancer types, YB-1 has been reported to promote cell proliferation [11,12], migration and invasion [13,14], as well as to inhibit apoptosis [15,16]. All of these mechanisms are in the repertoire of trophoblast activity and hence they are critical for adequate placental development. However, whether YB-1 affects trophoblast function remains to be elucidated. Supportive evidence for an involvement of YB-1 in placentation might be derived from the fact that, *in vitro*, YB-1 is able to induce matrix metalloproteinases (MMPs) [17,18]. These enzymes are capable of cleaving components of basal membranes and are therefore strongly involved in extensive tissue remodeling processes during placenta formation [19,20]. To follow up the idea of a potential participation of YB-1 in placentation and fetal development, we undertook the present study, with the aims of understanding whether YB-1 affects trophoblast functions and to evaluate whether trophoblast-derived YB-1 is crucial for placentation and fetal growth. For this, we first studied maternal and fetal parameters in YB-1 heterozygous intercrossings. After finding that fetal and placental growth as well as spiral artery (SA) remodeling was negatively affected by the partial or total absence of YB-1, we next generated trophoblast-specific YB-1-deficient mice to understand its impact in placenta physiology, fetal growth and fetal survival until day 14 of gestation. Placenta-specific YB-1 knockdown resulted in decreased placental areas, diameters, thicknesses and weights, but did not affect fetal weights. 

## 2. Materials and Methods

### 2.1. Mice 

Male and female C57BL/6 wild-type (WT, thus YB-1^+/+^) and heterozygous (HET, YB-1^+/−^) YB-1 mice were bred and maintained at our barrier facility. FVB Cyp19-Cre (5912 line) and floxed C57BL/6 YB-1 (YB-1^fl/fl^) mice were kindly provided by the groups of Prof. Leone (Ohio State University, Columbus, OH, USA) and Prof. Mertens (Magdeburg University, Magdeburg, Germany), respectively. Using these strains, we generated mice with trophoblast-specific YB-1 deficiency. FVB Cyp19-Cre females were mated to YB-1^fl/fl^ males and *vice versa.* As both mating combinations showed comparable results concerning their implantation and abortion rates, we focused our analyses on YB-1^fl/fl^-mated Cyp19-Cre females. Female FVB WT mice were purchased from Janvier, Le Genest-Saint-Isle, France. All mice were kept in a 12 h light/dark cycle at 22 ± 2 °C and an air humidity of 40–60%. Mice received water and food ad libitum. 

### 2.2. Experimental Design and Sample Collection

Animal experiments were performed according to the institutional guidelines upon ministerial approval (Landesverwaltungsamt Sachsen-Anhalt: 42502-2-1327 Uni MD). All experiments were conducted by authorized persons according to the Guide for Care and Use of Animals in Agriculture Research and Teaching. Eight- to eleven-week-old YB-1^+/−^ females were paired with YB-1^+/−^ males. YB-1^+/+^ females paired with YB-1^+/+^ males served as controls. In further experiments, FVB Cyp19-Cre or WT females were paired with YB-1^fl/fl^ males to obtain trophoblast-specific YB-1 knockdown animals or floxed controls, respectively. Females were checked twice a day for the appearance of a vaginal plug. Plug detection indicated gestation day (GD)0 of pregnancy. Serial *in vivo* ultrasound measurements were performed at GD5, 8, 10, 12 and 14. Animals were sacrificed at GD10, 12 or 14. Numbers of implantations and abortions were recorded. For SA analysis, one implantation per female was dissected at GD12 and fixed as explained elsewhere [21]. At GD12 and 14, fetal and placental weights were determined. 

### 2.3. High-Frequency Ultrasound Measurement

Serial ultrasound examinations were performed using the Vevo^®^ 2100 system (FujiFilm VisualSonics Inc., Amsterdam, The Netherlands) as described previously [22,23]. Briefly, mice were anesthetized with isoflurane, transferred and fixed to a heating platform and abdominal hair was removed with a depilatory cream. Eyes were protected from drying out by applying eye protection cream. Prewarmed ultrasound gel was applied on the abdomen, and ultrasound measurements were done with the transducer MS550D (22-55 MHz). Implantation areas (GD5, 8, 10, 12), as well as placental areas, thicknesses, and diameters (GD10, 12, 14) were measured in B-Mode. Color Doppler Mode and the Pulse-wave Doppler Mode were used to determine blood flow parameters in the maternal uterine arteries (GD5, 8, 10, 12, 14). With the Vevo LAB software, peak systolic velocities (PSVs) and end diastolic velocities (EDVs) were recorded (average of three measurements) and resistance indices (RI; RI  =  (PSV − EDV)/PSV) and pulsatility indices (PI; PI  =  (Velocity (V)max − Vmin)/Vmean) were calculated. During ultrasound measurement, ECG, body temperature and respiratory physiology of the mice were controlled throughout. 

### 2.4. Measurement of Fetal and Placental Weights

Fetal and placental weights were determined at GD12 and 14 using a scale (Kern & Sohn GmbH, Balingen, Germany, minimum: 0.02 g, sensitivity: 0.001 g). 

### 2.5. Histology

Implantations were fixed for 6 h in a 4% paraformaldehyde (PFA) solution containing 0.1 M saccharose (pH 7.4) by gently shaking at room temperature. Afterwards, they were stored overnight at 4 °C in 70% ethanol. The following day, implantations were cut into two halves, dehydrated through an ascending ethanol series, incubated in xylene and embedded in paraffin. Five-micrometer paraffin sections were stained with hematoxylin and eosin (H/E). H/E staining was performed as previously described [21].

### 2.6. Spiral Artery Analysis

SA parameters were analyzed in H/E-stained implantation sections at GD12. We collected one implantation site per female and subsequently analyzed 3–10 SAs, localized in the *decidua basalis* within the implantation site, via light microscope (Zeiss, Jena, Germany, 200× magnification). With the AxioVision software v4 (Zeiss), wall and lumen perimeters of SAs were measured to calculate wall and lumen diameters (diameter = circumference/π) and wall-to-lumen ratios (wall-to-lumen ratio = SA diameter/diameter lumen). The wall thickness was calculated ((SA diameter – lumen diameter)/2). Mean wall thicknesses and wall-to-lumen ratios were calculated for each female.

### 2.7. Statistics

GraphPad Prism 8.0 (Statcon GmbH, Witzenhausen, Germany) and SPSS Statistics 26.0 (IBM, Leipzig, Germany) were used for the statistical analysis of the data. Normality of the data was assessed with the D’ Agostino Pearson-Omnibus test. Data was analyzed by using the Mann–Whitney-*U* test (GraphPad Prism, Statcon GmbH) or a mixed linear model using the final test principle (SPSS, IBM, Leipzig, Germany). Data is presented as medians or means with standard deviation (SD). *p* values < 0.05 were considered to be statistically significant. 

## 3. Results

### 3.1. YB-1 Deficiency Negatively Affected Implantation and Fetal Growth 

To understand the participation of YB-1 in pregnancy outcome based on implantation and abortion rates, HET (YB-1^+/−^ x YB-1^+/−^) mating combinations were analyzed, with resulting WT (YB-1^+/+^), HET (YB-1^+/−^) and KO (YB-1^-/-^) placentas and fetuses. WT (YB-1^+/+^ x YB-1^+/+^) combinations served as controls. Neither partial nor total deficiency in placental tissue affected the number of implantations or abortion (Table 1).

**Figure 1:**
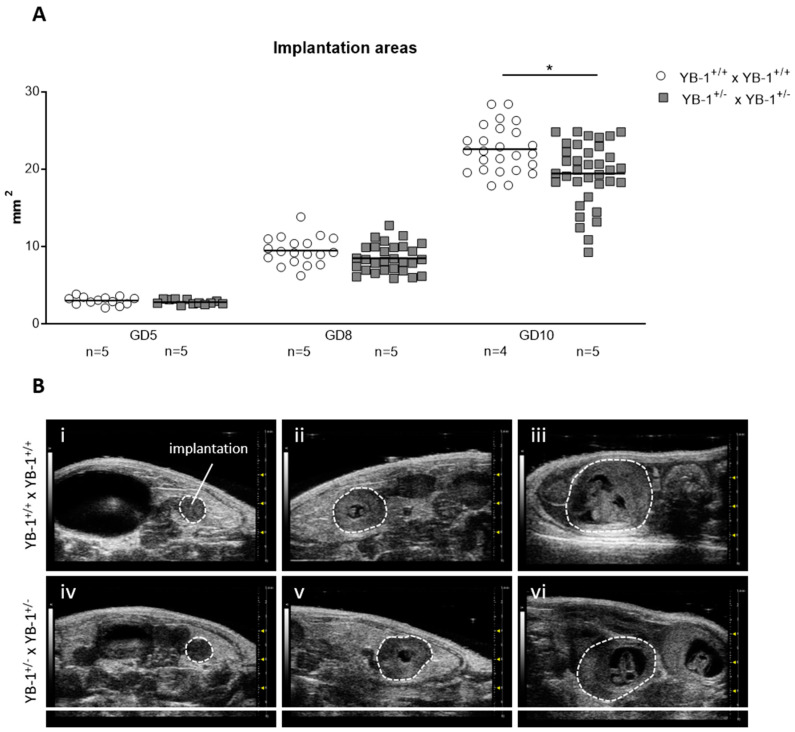
Decreased implantation areas in pregnant YB-1^+/−^ females compared to YB-1^+/+^ females at GD10. (**A**) Implantation areas of single implantations of YB-1^+/+^ × YB-1^+/+^ (*n* = 4–5) or YB-1^+/−^ × YB-1^+/−^ (*n* = 5) matings at GD5, 8, and 10. The data is presented as medians showing individual values for each implantation site. Statistical differences between both mating combinations per time point were determined by using a mixed linear model using the final test principle (* *p* < 0.05). (**B**) Representative 2D greyscale ultrasound images at GD5 (i, iv), 8 (ii, v), and 10 (iii, vi) from YB-1 WT (i–iii) and YB-1 HET (iv–vi) matings are shown.

Next, serial high-frequency ultrasound examinations were performed during gestation of YB-1^+/+^ and YB-1^+/−^ females to follow fetal growth *in vivo*. For this, the size of whole implantations were analyzed. At GD5 and GD8, no differences were detected among the maternal genotypes. This changed as pregnancy advanced, with implantation areas of YB-1^+/−^ females being significantly reduced (*p* < 0.05) at GD10 compared to YB-1^+/+^ control females at the same gestational point (Figure 1A). Representative 2D greyscale images showing whole implantations from YB-1^+/+^ (i–iii) or YB-1^+/−^ (iv–vi) females are displayed in Figure 1B. 

### 3.2. YB-1 Deficiency Altered Fetal and Placental Parameters 

**Figure 2:**
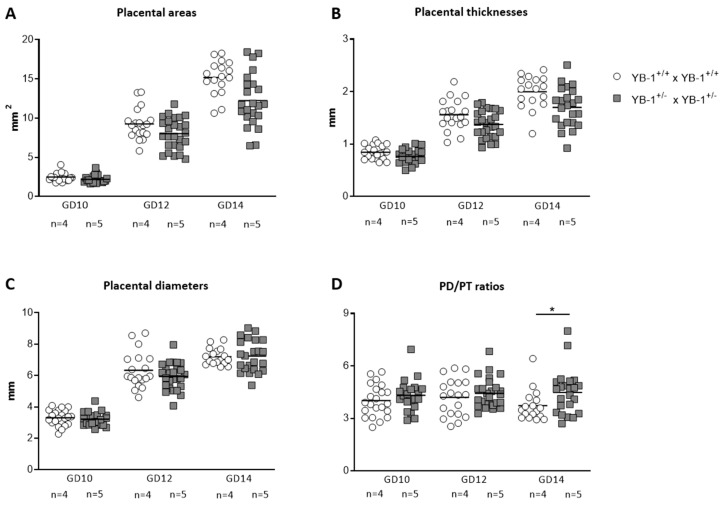
Significant increased placental diameter/thickness ratios in pregnant YB-1^+/−^ compared to YB-1^+/+^ females at GD14. Placental areas (**A**), thicknesses (**B**), diameters (**C**) and diameter/thickness (PD/PT) ratios (**D**) of YB-1^+/+^ x YB-1^+/+^ (*n* = 4) and YB-1^+/−^ x YB-1^+/−^ (*n* = 5) matings are displayed at GD10, 12 and 14. Placental parameters are presented as medians showing individual values for each placenta. Statistical differences were determined by using a mixed linear model using the final test principle (* *p* < 0.05).

Diminished implantation areas as observed in fetuses from YB-1^+/−^ females might be a consequence of impaired placental and/or fetal growth. To analyze whether YB-1 is pivotal for placental growth, serial ultrasound measurements were performed to assess the following placental parameters: area, thickness and diameter (Figure S1). All three placental parameters (Figure 2A–C) were comparable between YB-1 WT and HET mating combinations for the analyzed time points GD10, 12 and 14. However, placental diameter/thickness ratios that are strong indicators of placental insufficiency, were significantly increased (*p* < 0.05) in YB-1^+/−^ females compared to YB-1^+/+^ females (Figure 2D). 

**Figure 3:**
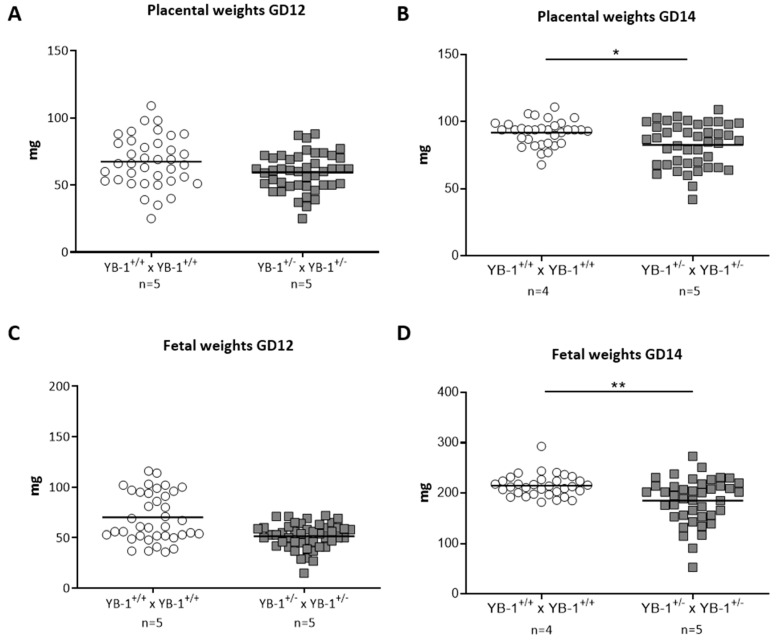
Significant decreased placental and fetal weights in YB-1^+/−^ females compared to YB-1^+/+^ females at GD14. Placental weights (**A**,**B**) and fetal weights (**C**,**D**) from the progeny of YB-1^+/+^ × YB-1^+/+^ (*n* = 4–5) or YB-1^+/−^ × YB-1^+/−^ (*n* = 5) matings are shown at GD12 and 14. Data is presented as medians showing individual values for each fetus/placenta. Statistical differences were determined by using a mixed linear model using the final test principle (* *p* < 0.05; ** *p* < 0.01).

While placental and fetal weights were comparable at GD12 (Figure 3A,C), YB-1 HET females presented significantly reduced placental and fetal weights at GD14 compared to their YB-1^+/+^ counterparts (Figure 3B,D). 

### 3.3. Placental YB-1 Deficiency Resulted in Impaired Remodeling of Uterine Spiral Arteries without Affecting Uterine Artery Flow Parameters

**Figure 4:**
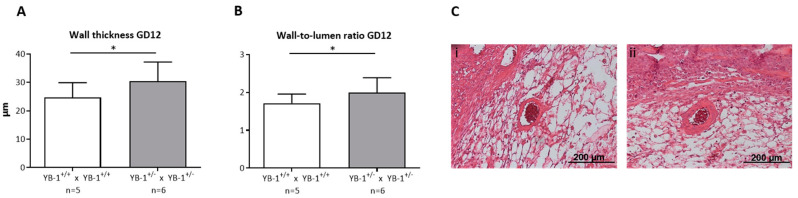
Impaired SA remodeling in YB-1^+/−^ females compared to YB-1^+/+^ females at GD12. Wall thicknesses (**A**) and wall-to-lumen ratios (**B**) of YB-1^+/+^ x YB-1^+/+^ (*n* = 5) or YB-1^+/−^ x YB-1^+/−^ (*n* = 6) matings are displayed at GD12. Results are presented as means with standard deviation (SD) and analyzed by using a mixed linear model using the final test principle (* *p* < 0.05). (**C**) Representative hematoxylin/eosin-stained slides of implantations showing uterine spiral arteries (SAs) in the *decidua basalis* of YB-1^+/+^ (i) and YB-1^+/−^ (ii) females at GD12 (scale bar = 200 µm).

Next, we focused on the analysis of the remodeling process that takes place during placentation in the maternal uterus and is crucial for fetal growth. During this process, uterine SAs are modified from low-flow, high-resistance to high-flow, low-resistance vessels capable of fulfilling the demands of the developing fetus [24]. YB-1^+/−^ females showed insufficiently remodeled SAs when compared to YB-1^+/+^ females. More precisely, as a signature of inadequately and incompletely remodeled SA, there was a significantly higher (*p* < 0.05) wall thickness (Figure 4A), resulting in a higher (*p* < 0.05) and thus pathologic wall-to-lumen ratio (Figure 4B) in YB-1^+/−^ females compared to WT control females. Representative pictures of WT matings (i) and HET matings (ii) are shown in Figure 4C. As a consequence of insufficiently remodeled SAs, hemodynamic alterations can be expected in these vessels. Accordingly, the peak systolic and end diastolic velocity (Figure S2) were determined to calculate the PI and RI at GD10, 12, and 14 (Figure S2B,C). However, both indices indicative for the uterine blood flow were not changed when YB-1 was reduced. 

### 3.4. Trophoblast-Specific YB-1 Deficiency Negatively Affected Fetal and Placental Parameters with a Phenotype of Decreased Placental Weight

Next, we wondered whether YB-1 expressed in trophoblasts regulates critical functions in fetal and placental growth. To answer this relevant question, we generated trophoblast-specific YB-1-deficient mice by mating Cyp19-Cre females with YB-1^fl/fl^- males. Controls included YB-1^fl/fl^-mated WT females. Our results revealed that trophoblast-specific YB-1 ablation had no effect on implantation number and fetal survival as analyzed in pregnant females at GD10, 12 and 14 (Table 2). 

**Figure 5:**
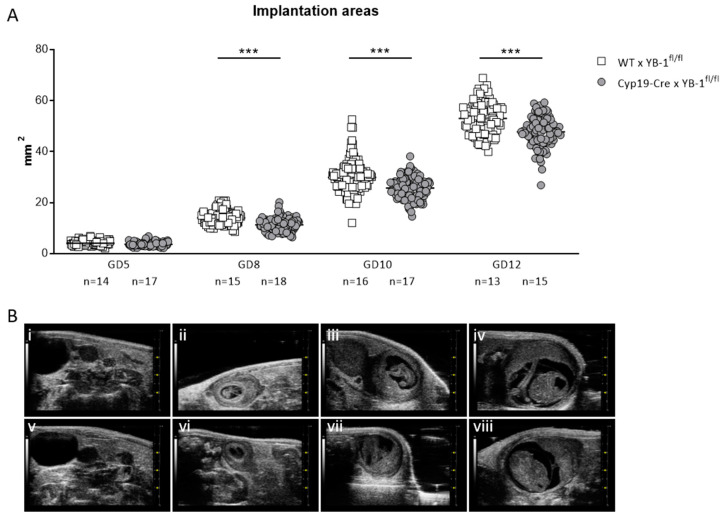
Decreased implantation areas in YB-1^fl/fl^-mated Cyp19-Cre females compared to YB-1^fl/fl^-mated WT females from GD8 onwards. (**A**) Implantation areas of single implantations of Cyp19-Cre x YB-1^fl/fl^ (*n* = 6–16) or WT x YB-1^fl/fl^ (*n* = 6–18) matings at GD5, 8, 10 and 12. Results are presented as means showing individual values for each implantation. Statistical differences were determined by using a mixed linear model using the final test principle (*** *p* < 0.001). (**B**) Representative 2D greyscale ultrasound images at GD5 (i, v), 8 (ii, vi), 10 (iii, vii) and 12 (iv, viii) from WT × YB-1^fl/fl^ (i–iv) and Cyp19-Cre × YB-1^fl/fl^ (v–viii) matings.

Even though the number of implantation and surviving fetuses did not seem to be affected, serial high-frequency ultrasound examinations revealed that the absence of trophoblast-derived YB-1 resulted in significantly reduced (*p* < 0.001) implantation areas at GD8, 10 and 12 (Figure 5A). Representative 2D greyscale images are shown of WT (i–iv) and Cyp19-Cre (v–viii) females in Figure 5B. 

**Figure 6:**
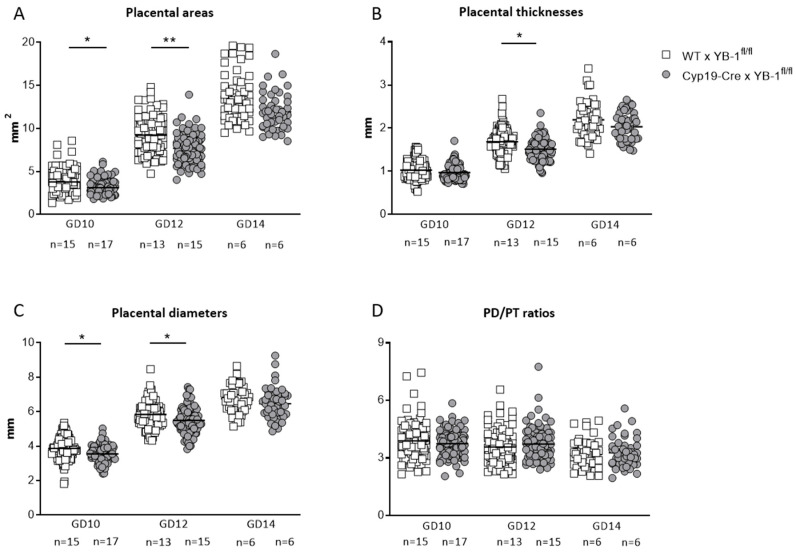
Altered placental parameters in YB-1^fl/fl^-mated Cyp19-Cre females compared to YB-1^fl/fl^-mated WT females at GD14. Placental areas (**A**), thicknesses (**B**), diameters (**C**) and diameter/thickness (PD/PT) ratios (**D**) of Cyp19-Cre x YB-1^fl/fl^ (*n* = 6–15) or WT × YB-1^fl/fl^ (*n* = 6–17) matings are displayed at GD10, 12 and 14. Placental parameters are presented as means showing individual values for each placenta. Statistical differences were determined by using a mixed linear model using the final test principle (* *p* < 0.05; ** *p* < 0.01).

Moreover, placental areas (*p* < 0.05, *p* < 0.01) and placental diameters (*p* < 0.05) were significantly decreased in Cyp19-Cre females mated to YB-1^fl/fl^ males compared to WT females mated to YB-1^fl/fl^ males at GD10 and 12 (Figure 6A,C). Accordingly, placental thicknesses were significantly diminished (*p* < 0.05) at GD12 (Figure 6B), regardless of the fact that placental diameter/thickness ratios were not significantly affected by trophoblastic YB-1 ablation (Figure 6D).

**Figure 7:**
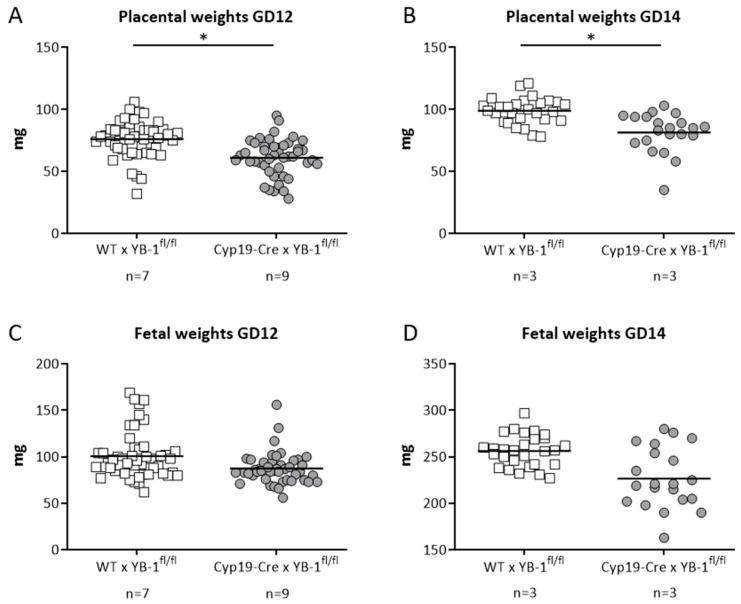
Significantly decreased placental weights in in YB-1^fl/fl^-mated Cyp19-Cre females compared to YB-1^fl/fl^-mated WT females at GD12 and 14. Placental weights (**A**,**B**) and fetal weights (**C**,**D**) from progeny of Cyp19-Cre × YB-1^fl/fl^ (*n* = 3–7) or WT × YB-1^fl/fl^ (*n* = 3–9) matings are shown at GD12 and 14. Data is presented as means showing individual values for each placenta/fetus. Statistical differences were determined by using a mixed linear model using the final test principle (* *p* < 0.05).

In line with the results obtained in heterozygous combinations, YB-1 deficiency in trophoblast cells did not alter uterine artery flow parameters. More precisely, uterine artery PI and RI were comparable between both mating combinations at GD5, 8, 10, 12 and 14 (Figure S3B,C). Both indices were calculated based on the determination of the peak systolic and end diastolic velocities (Figure S3A). Finally, we analyzed whether trophoblast-derived YB-1 is pivotal for placental and fetal growth. For this, fetal and placental weights were recorded at GD12 and 14. Placental weights were significantly lower (*p* < 0.05) in YB-1^fl/fl^-mated Cyp19-Cre females when compared to YB-1^fl/fl^ -mated WT females at GD12 and 14 (Figure 7A,B) with no significant differences in fetal weights at the same gestation day (Figure 7C,D). 

## 4. Discussion

A myriad of functions have been attributed to YB-1. Among these, YB-1 plays a major role in cancer development and progression due to its ability to support the proliferation, invasion and metastasis of various tumor cells [12,25,26,27]. Moreover, this cold shock protein is also relevant for the regulation of inflammatory responses due to the transcriptional and translational regulation of inflammatory mediators [28,29,30]. Previous data by Lu and colleagues as well as Uchiumi and colleagues also strongly suggest that YB-1 may be relevant for pregnancy outcome or fetal wellbeing [8,9]. YB-1 HET intercrossings resulted in a significant growth retardation in YB-1-deficient fetuses when compared to their YB-1-half-sufficient and -sufficient littermates from mid-gestation onwards. YB-1 KO fetuses showed severe malformations in different organ systems and eventually died perinatally [8,9]. As the nonviability of YB-1 KO progeny hinders experiments employing homozygous intercrossing combinations, we paired YB-1 heterozygous mice and compared fetal and maternal parameters with those of YB-1 WT matings. Our results strongly suggest that YB-1 absence does not interfere with the ability of the embryos to implant into the maternal endometrium. Obviously, embryonic lethality seems to be restricted to later gestational time points, as proposed in previous studies [8,9]. However, this does not rule out the possibility that YB-1 affects early pregnancy mechanisms whose readout appears later and derives from fetal impairment. 

Despite an apparently undisturbed implantation, fetal weights were reduced in YB-1 HET mating combinations when compared to YB-1 WT controls at GD14. Moreover, we detected significant smaller implantation areas in YB-1 HET combinations compared to YB-1 WT controls already at GD10. As our intention was to follow up implantation areas, placental parameters and uterine flow parameters in the same pregnant mouse from GD5 to GD14 by sequential ultrasound measurements, in this set of experiments, we did not sacrifice mice earlier than GD14. This kind of analysis impeded the genotypic determination of the individual implantations at GD10. Thus, we assume that implantations of significantly reduced size are the ones completely lacking YB-1, and further evaluations are required to confirm this assumption. As YB-1 expression is strong in trophoblasts, we next followed the strategy of generating trophoblast-specific YB-1-deficient mice. 

Fetal growth mainly depends on adequate placental development and function, which are usually reflected in placental size, shape and histological structure. Consequently, impairments in placental development resulting in decreased placental surface areas, decreased placental diameters and decreased placental weights are associated with fetal growth retardation [31,32,33] and usually lead to adverse pregnancy outcomes or fetal/neonatal perinatal mortality and morbidity. We assessed placental parameters at different GDs and found a significant upregulation in the placental diameter/thickness ratios as well as a significant downregulation in the placental weights in YB-1 HET matings compared to YB-1 WT matings at GD14. Given the fact that placental disc thickness marks the extent of the maternal fetal exchange area, an increased placental diameter/thickness ratio because of a decreased placental thickness is indicative for fetal malnutrition and thus a lower birth weight [34]. Moreover, small placental areas and low placental weights are usually associated with higher uterine PI and RI [35] that may result in reduced placental perfusion and subsequently in insufficient nutrient supply to the fetus. Therefore, we analyzed uterine flow parameters by high-frequency ultrasound during pregnancy. Although in our experimental settings, uterine flow parameters were not altered in YB-1 HET matings, histological analysis of SA remodeling parameters displayed a picture of inadequate SA remodeling. Placentas from heterozygote mating combinations had increased, thus disturbed, SA wall-to-lumen ratios. Causative for this insufficient SA remodeling process are often perturbed mechanisms from both the maternal and the fetal side. Hence, in addition to a potential contribution of maternal YB-1 to the SA remodeling process, we assumed that poor trophoblast function characterized by the inability of these cells to proliferate, migrate and/or invade the arteries may be responsible for the observed phenotype [36]. Thus, we wondered whether a specific ablation of YB-1 in trophoblast cells would similarly affect fetal and placental outcomes as observed in the YB-1 HET intercrossings. To study a trophoblast-specific deletion of YB-1, we generated Cyp19Cre/YB-1^fl/fl^ mice, whose placentas are YB-1-deficient. In the Cyp19Cre mice, the Cre transgene is driven by placenta-specific regulatory sequences from the human Cyp19 gene. Cre expression was confirmed in trophoblast stem cells in the extraembryonic ectoderm and the ectoplacental cone at E6.5 and E8.5 [37]. Several research groups have used Cyp19Cre mice for the placenta-specific deletion of distinct genes to study their participation in placenta formation and pregnancy outcomes [38,39,40,41]. However, all placentas still contained one YB-1 WT allele, resulting in a reduction of placental YB-1 expression by 50%. This is in accordance with results obtained by a study in which the authors used Cyp19Cre/IL-6Rα mice to specifically delete IL-6Rα in the placenta. mRNA and protein expression analyses revealed a 50% reduction of placental IL-6Rα [42]. After placenta-specific knockdown of YB-1, impairments in fetal and placental parameters occurred even earlier than in YB-1 HET mating combinations. Significant reductions in implantations areas were already visible at GD8, and the first signs of altered placental parameters were evident from GD10 onwards. Unexpectedly, fetal weights were not significantly diminished after placenta-specific YB-1 ablation at GD14, which contrasts with our observations made in YB-1 HET combinations. This might be explained by the fact that fetuses of Cyp19Cre/YB-1^fl/fl^ matings are not directly affected by placental YB-1 knockdown and significantly reduced fetal weights in YB-1 HET matings are the consequence of missing YB-1 in the placenta and in the fetus. Moreover, we have to admit that in our mating combination, a complete absence of YB-1 in the placenta could not be achieved, and this might also explain the insignificant diminution in fetal weights. In accordance with our results obtained in YB-1 HET matings, the number of implantations and abortion rates was similar in Cyp19Cre/YB-1^fl/fl^ matings.

Data on cold shock proteins in general and YB-1 in particular are scarce for the field of placentation, and until now, we could only speculate about YB-1-driven mechanisms in placentation. Extracellular YB-1 was proven to serve as a noncanonical ligand for receptor Notch-3 [43], and a role for Notch3 signaling in trophoblast migration and invasion was suggested [44,45]. Further research is needed to confirm YB-1 autocrine effects on trophoblast function through this pathway. As trophoblast cells and cancer cells share common functional features, including their potential to proliferate, migrate, invade, and to induce vascularization [46], we firmly believe that YB-1 is an interesting candidate to follow up in the research area of pregnancy and more precisely for the events around placentation. Our data show that whole-body heterozygous mice combination results in impaired placentation and fetal growth. A more elegant approach employing trophoblast-specific YB-1 knock-down refines the finding by showing suboptimal developed placentas. In cancer cells, YB-1 has been shown to activate the expression of several genes important for cell proliferation, such as DNA polymerase α, cyclins, PIK3CA, EGFR and HER-2 [47], with some of these molecules being implicated in trophoblast proliferation [48,49,50]. Furthermore, YB-1 promotes cancer migration and invasion by activating the metalloproteinases MMP2 and MT1 [17,18]. MMPs are well known for their participation in trophoblast-mediated functions [51], and we propose that YB-1 supports placentation through the modulation of MMPs. Finally, YB-1 might also play a critical role for placentation-associated angiogenesis by inducing the expression of proangiogenic genes [47,52]. However, the involvement of YB-1 in these placentation pathways remains speculative and is the focus of ongoing studies.

## 5. Conclusions

In summary, our data show the importance of YB-1 for intrauterine embryonic/fetal and placental development from early gestational stages. Moreover, our study provides the first evidence for the relevance of trophoblast-specific YB-1 to placentation. Future studies will dissect the underlying pathways and determine whether these pathways are relevant for patients whose babies are born growth-retarded. 

## Figures and Tables

**Table 1:** YB-1 deficiency did not affect implantation and abortion rates.

Measurement	YB-1^+/+^ × YB-1^+/+^	YB-1^+/−^ × YB-1^+/−^
Number of implantations	10 (3–12)	10 (9–11)
Number of abortions	2 (0–3)	1 (0–3)
Abortion rate (%)	18.13 (0.00–100)	10.56 (10–27.27)

Analysis included YB-1^+/+^ females (*n* = 10) and YB-1^+/−^ females (*n* = 10), mated to YB-1^+/+^ or YB-1^+/−^ males, respectively. Data sets represent combined data from GD12 (*n* = 5 per mating combination) and GD14 (*n* = 5 per mating combination). Data is shown as the medians plus range. Statistical analysis was performed with the Mann–Whitney-*U* test.

**Table 2:** YB-1 deficiency in trophoblast cells did not affect implantation and abortion rates.

Measurement	WT × YB-1^fl/fl^	Cyp19-Cre × YB-1^fl/fl^
Number of implantations	11 (1–13)	9 (2–14)
Number of abortions	0 (0–3)	1 (0–3)
Abortion rate (%)	0.00 (0.00–23.08)	11.11 (0.00–50.00)

Analysis included WT females (*n*=13) and Cyp19-Cre females (*n* = 15) mated to YB-1^fl/fl^ males. Data sets represent combined data from GD10 (*n* = 3 per mating combination), GD12 (*n* = 7 or 9 per mating combination) and GD14 (*n* = 3 per mating combination). Data is shown as medians plus range. Statistical analysis was performed with the Mann–Whitney-*U* test.

## Supplementary Materials

The following are available online at https://www.mdpi.com/2073-4409/9/9/1942/s1, Figure S1: Representative 2D greyscale ultrasound image of one implantation site showing placental area, diameter and thickness; Figure S2: No alterations of uterine artery (UA) flow parameters in YB-1^+/−^ females compared to YB-1^+/+^ females at GD10, 12 and 14; Figure S3: No alterations of uterine artery (UA) flow parameters in YB-1^fl/fl^-mated Cyp19-Cre females compared to YB-1^fl/fl^-mated WT females at GD5, 8, 10, 12 and 14.
