# Supplementary material for: Y-Box Binding Protein 1 Expression in Trophoblast Cells Promotes Fetal and Placental Development
Source: Cells. 2020 Aug 22;9(9):1942. doi: 10.3390/cells9091942 (PMC7563187; doi:10.3390/cells9091942)
Supplement: Supplementary file 1 [file cells-09-01942-s001.pdf]

cells

MDPI

Article

Y-Box Binding Protein 1 Expression in Trophoblast Cells Promotes Fetal and Placental Development

Nicole Meyer [1,2,†], Anne Schumacher [1,2,†], Urs Coenen [1], Katja Woidacki [1], Hannah Schmidt [1], Jonathan A. Lindquist [3], Peter R. Mertens [3] and Ana C. Zenclussen [1,2,*]

1 Experimental Obstetrics and Gynecology, Medical Faculty, Otto-von-Guericke University, 39108 Magdeburg, Germany; nicole.meyer@med.ovgu.de (N.M.); anne.schumacher@med.ovgu.de (A.S.); urs.coenen@outlook.com (U.C.); katja.woidacki@med.ovgu.de (K.W.); Hannah.Schmidt.HS@posteo.de (H.S.)

2 Department of Environmental Immunology, Helmholtz Centre for Environmental Research, 04318 Leipzig, Germany

3 Department of Nephrology and Hypertension, Diabetes and Endocrinology, Otto-von-Guericke University, 39120 Magdeburg, Germany; jon.lindquist@med.ovgu.de (J.A.L.); peter.mertens@med.ovgu.de (P.R.M.)

* Correspondence: ana.zenclussen@ufz.de; Tel.: +49-341-235-1265

† These authors contributed equally to this work.

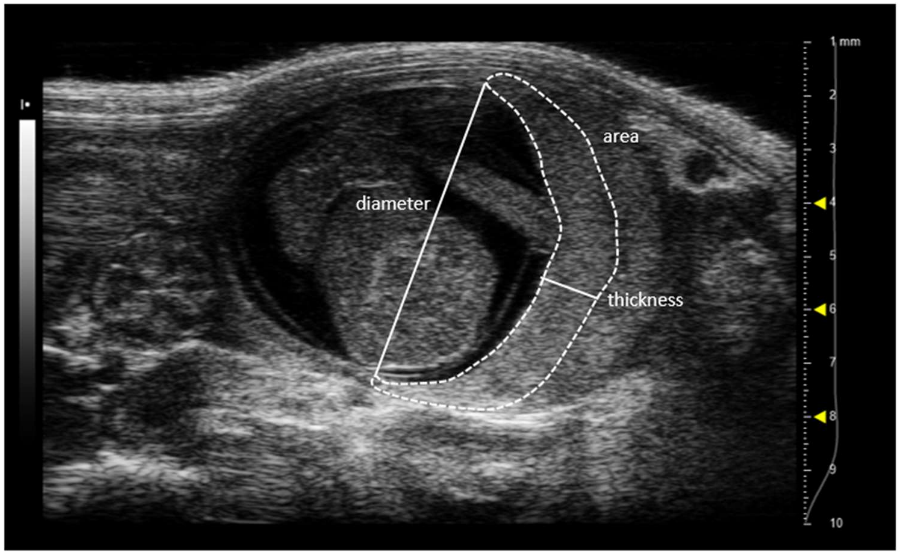

Figure S1. Representative 2D greyscale ultrasound image of one implantation site showing placental area, diameter and thickness.

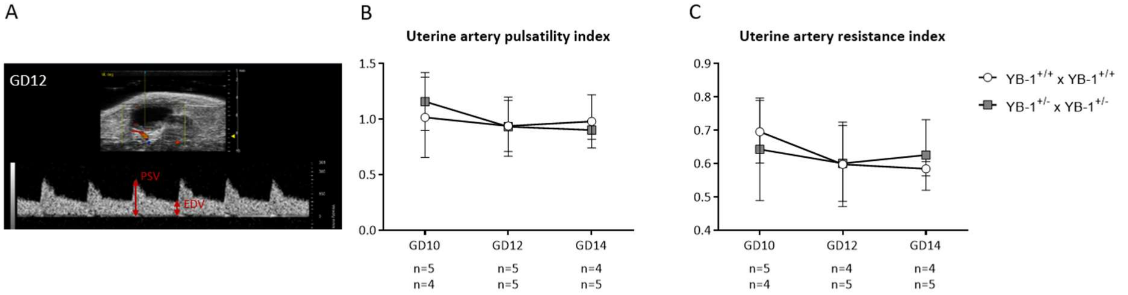

Figure S2. No alterations of uterine artery (UA) flow parameters in YB-1$^{+/-}$ females compared to YB-1$^{+/+}$ females at GD10, 12 and 14. **(A)** Representative 2D greyscale ultrasound image of the maternal UA visualizing the peak systolic velocity (PSV), the end diastolic velocity (EDV) as well as the frequency. UA pulsatility index (PI) **(B)** and resistance index (RI) **(C)** of YB-1$^{+/+}$ × YB-1$^{+/+}$ (n = 4–5) or YB-1$^{+/-}$ × YB-1$^{+/-}$ (n = 5) matings at GD10, 12, and 14. The data is presented as means plus standard deviation (SD). Statistical differences between both mating combinations per time point were determined by using the Mann-Whitney-*U* test.

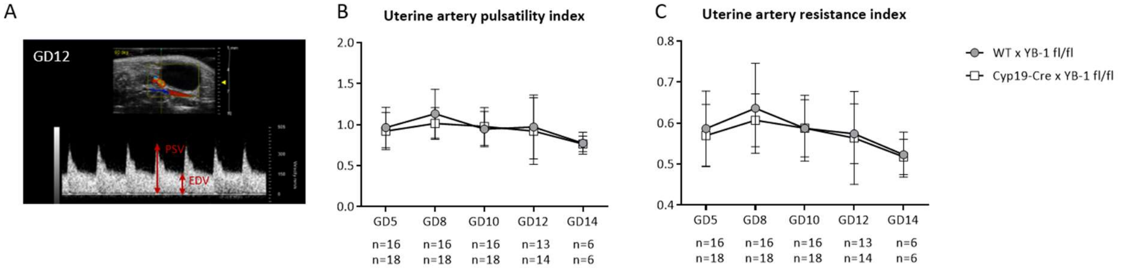

Figure S3. No alterations of uterine artery (UA) flow parameters in YB-1$^{fl/fl}$-mated Cyp19-Cre females compared to YB-1$^{fl/fl}$-mated WT females at GD5, 8, 10, 12 and 14. **(A)** Representative 2D greyscale ultrasound image of the maternal UA visualizing the peak systolic velocity (PSV), the end diastolic velocity (EDV) as well as the frequency. UA pulsatility index (PI) **(B)** and resistance index (RI) **(C)** of YB-1$^{fl/fl}$-mated Cyp19-Cre females (n = 6–16) or YB-1$^{fl/fl}$-mated WT females (n = 6-18) matings at GD5, 8, 10, 12, and 14. The data is presented as means plus standard deviation (SD). Statistical differences between both mating combinations per time point were determined by using the Mann-Whitney-*U* test.
